# In Situ Pyrolysis of Oil Shale with the Copper-Amine Complex as an Efficient Catalyst

**DOI:** 10.1021/acsomega.6c05797

**Published:** 2026-09-25

**Authors:** Lianhua Hou, Chen Wang, Jing Chen, Bo Sun, Ziyi Xu, Baojian Shen, Chunming Xu, Xiaochun Zhu

**Affiliations:** † 205361 Research Institute of Petroleum Exploration and Development, PetroChina, Beijing 100083, P. R. China; ‡ State Key Laboratory of Heavy Oil Processing, College of Chemical Engineering and Environment, 74537 China University of Petroleum Beijing, Beijing 102249, P. R. China

## Abstract

The Triassic Chang 7 shale in the Ordos Basin is recognized as an important supplementary resource alongside conventional petroleum reserves. Nevertheless, conventional techniques cannot achieve cost-effective production. In this study, a copper-based catalyst, copper-tetraethylenepentamine (Cu-TEPA), was synthesized and its catalytic performance was evaluated via shale pyrolysis in a sealed system under fixed experimental conditions: a thermal reaction temperature of 450 °C, a constant heating duration of 3 h, and a Cu-TEPA-to-raw shale mass ratio of 0.1 wt %. The introduction of Cu-TEPA resulted in a remarkable enhancement of pyrolysis efficiency and the hydrocarbon recovery rate; the shale oil yield increased significantly by 33.33% as compared with the noncatalytic pyrolysis. Simultaneously, Cu-TEPA exhibited excellent catalytic performance in heteroatom removal, achieving removal rates of 32.8% for sulfur atoms and 55.1% for nitrogen atoms, which significantly improved the quality of the product oil. The copper-amine complex catalyst shows great potential for cost-effective in situ shale oil production.

## Graphical abstract

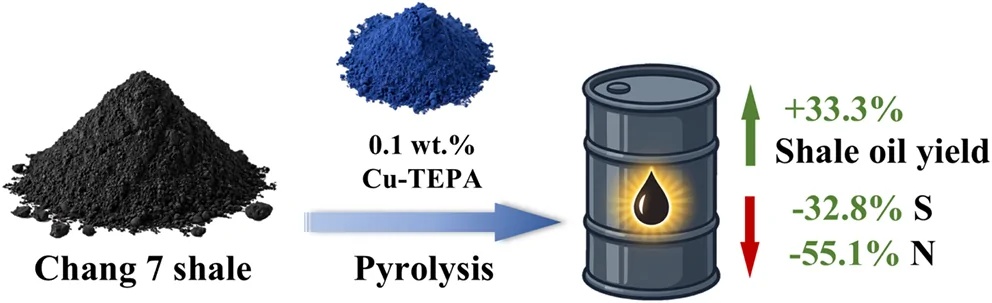

## 1 Introduction

The discrepancy between energy supply and demand has intensified the search for alternative energy sources.^1–4^ Recoverable reserves of unconventional petroleum resources (e.g., shale oil and heavy oil) exceed those of conventional fossil resources (crude oil, natural gas, and coal). China possesses particularly abundant lacustrine oil shale resources, estimated at 7.2 × 10^11^ tons.^5,6^ However, the majority of these oil shale resources exhibit relatively low thermal maturity,^7–10^ resulting in low kerogen conversion and consequently low hydrocarbon recovery. The highly efficient exploitation of oil shale has the potential to become the most important supplement to petroleum and natural gas in the future.^11–13^

Dozens of technologies have been established for pyrolysis of kerogen in shale.^14^ The pyrolysis technologies of oil shale mainly include ex-situ retorting on the ground^15,16^ and in situ pyrolysis underground.^17,18^ The ex-situ retorting process directly damages the surface ecology,^19–21^ occupies a large area of surface land, produces semicoke waste, and causes emission pollution, which limits its large-scale utilization.^22,23^ In contrast, the in situ pyrolysis involves heating underground, converting kerogen into light oil and natural gas directly within the subsurface reservoir. Techniques such as steam injection, electrical heating, combustion, or radiative heating could facilitate this conversion, enabling the resulting fluids to be produced through conventional wells.^24,25^ This method has the advantages of environmental friendliness and low cost, representing the core direction for future oil shale resource development.^26^ At present, more than 20 in situ conversion technologies have been developed, such as the In situ Conversion Process (ICP) from Shell,^27,28^ Electrofrac technology of ExxonMobil,^8,29^ CCR technology, CRUSH technology,^30^ and underground local chemical in situ conversion technology (Jilin University).^31^ In China, the oil shale (Chang 7 shale) within the Ordos Basin and the Cretaceous Nenjiang Formation in the Songliao Basin are widely recognized as prime candidates for in situ pyrolysis technology.^32,33^ The recoverable resources in the shales were estimated to be over 70 billion tonnes of product oil and over 65 trillion cubic meters of natural gas.^34^ However, in situ pyrolysis process usually takes 3 to 5 years, leading to massive energy consumption and causing secondary cracking of the product oil.^35^

Catalysts play an important role in regulating product selectivity and reducing activation energy of various reactions. Previous studies reported that the transition metal salts and sulfides,^36,37^ molecular sieves,^38–40^ minerals,^41–43^ and porous organic polymers^44,45^ were able to reduce the activation energy associated with oil shale pyrolysis and improve the yield and quality of product oil. However, the nanometer-scale pores of low maturity shale severely hinder the diffusion of solid catalysts in the subsurface reservoir.^46^ To enhance the performance and utilization of catalyst underground, we propose a method for pyrolyzing oil shale with a copper-based catalyst Cu-TEPA, in which the divalent copper ions are chelated by amino-containing organic ligands such as tetraethylenepentamine. The dispersion of metal salt catalysts is often hindered by the adsorption, ion-exchange, or complexation in the subsurface. The formation of metal–ligand complexes would delocalize the charge on the metal center, weakening its electrostatic interaction with mineral surfaces. This reduction in adsorption enhances the mobility and transport of metal species in subsurface nanoporous networks, thereby enhancing the delivery of metal active sites to the organic kerogen. In this study, the in situ catalytic pyrolysis process was conducted using a closed system with Chang 7 shale. The impact of the catalyst on shale pyrolysis was investigated and the product yields and oil/gas composition were analyzed in detail. The salient findings illustrate the potential of Cu-TEPA for practical application in the in situ conversion of oil shale.

## 2 Materials and Methods

### 2.1 Materials

Tetraethylenepentamine (C_8_H_23_N_5_, ≥90%) was obtained from Beijing Yili Fine Chemicals Co., Ltd. Copper sulfate pentahydrate (CuSO_4_·5H_2_O, ≥99%) was purchased from Shanghai Aladdin Biochemical Technology Co., Ltd. Dichloromethane (CH_2_Cl_2_, 99.5%) was purchased from Beijing Tongguang Fine Chemicals Company. Ethanol (C_2_H_6_O, ≥99.7%) was received from Shanghai Aladdin Biochemical Technology Co., Ltd. The shale samples were obtained from the seventh member of the Yanchang Formation (Chang 7 shale) of the Triassic System in Ordos Basin, and their basic physical properties and proximate analysis results are presented in Tables S1–S3.

### 2.2 Synthesis of the Cu-TEPA Catalyst

Cu-TEPA was synthesized according to a published procedure.^47^ In a typical synthesis, 1.25 g of copper sulfate pentahydrate (CuSO_4_·5H_2_O) was dissolved in 50 mL of ultrapure water under continuous stirring. The solution was sonicated to ensure complete dissolution. Afterward, 0.94 g of tetraethylenepentamine (TEPA) (molar ratio of CuSO_4_·5H_2_O:TEPA = 1:1) was slowly added into the copper sulfate solution under a nitrogen atmosphere. To maintain an oxygen-free environment, the reaction vessel was sealed. The mixture was continuously stirred at ambient temperature for 4 h. Finally, the resulting solution was freeze-dried and ground into a fine powder, which was named as Cu-TEPA. Figure 1 illustrates the synthesis approach of the Cu-TEPA catalyst.

**Figure 1:**
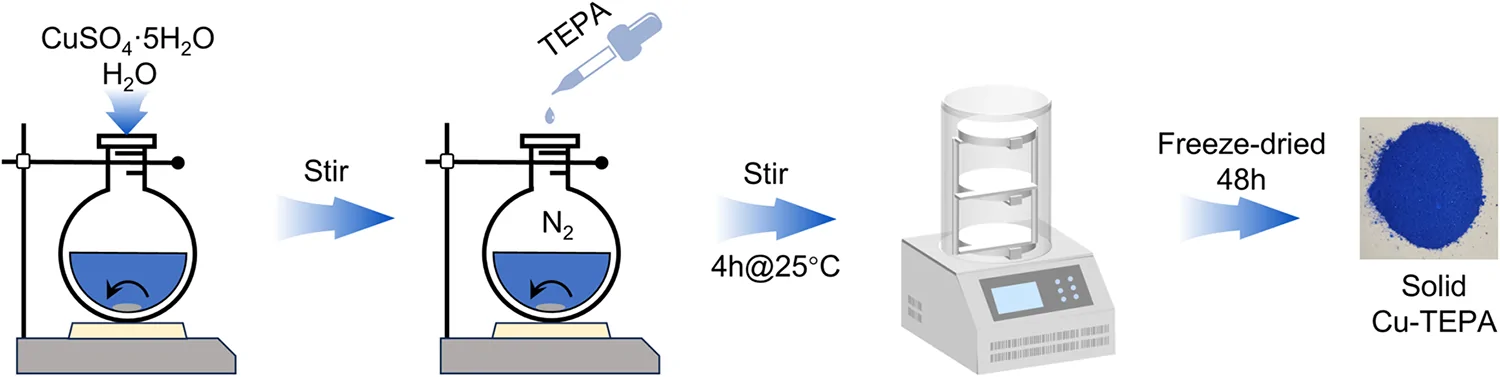
Synthesis approach of the Cu-TEPA catalyst.

### 2.3 Characterization

PANalytical X’Pert Powder X-ray diffractometer (XRD) with Cu–Kα radiation (λ = 0.15 nm) and a scanning range of 2θ = 5–85° was used for the phase analysis of Cu-TEPA. A Zeiss Gemini SEM 300 scanning electron microscope was used to observe the Cu-TEPA catalyst. Thermogravimetric analysis (TGA) was carried out using a Mettler Toledo Thermal Analysis instrument; the temperature increased from room temperature to 800 °C at a heating rate of 10 °C/min. FT-IR spectra were acquired over 4000–400 cm^–1^ on a Thermo Fisher Nicolet IS10 instrument. The elemental composition and valence states of the catalysts were determined using a Thermo Fisher Nexsa X-ray photoelectron spectrometer (XPS) with a monochromatic Al *K*α X-ray source, charge compensation by an electron flood gun for powder samples (pressed onto double-sided carbon tape), binding-energy calibration against the adventitious C 1s peak at 284.8 eV, and peak fitting using a Shirley-type background. Semicoke samples were analyzed for radical concentration on a Bruker A300 electron paramagnetic resonance (EPR) spectrometer; measurements were performed at room temperature using a sample tube with a diameter of 5 mm. The microwave power was set to 19.71 mW at a frequency of 9.853 GHz. Scan width was 200 G, the time constant was 10.24 ms, and the scan time was set to 60 s. The modulation frequency was set to 100 kHz with a modulation amplitude of 1 G.

### 2.4 Pyrolysis Experiments

The pyrolysis of Chang 7 shale was conducted in a closed system. Cu-TEPA (0.03 g) was dissolved in deionized water. Afterward, 30 g of Chang 7 shale was blended with the catalyst solution and subsequently freeze-dried to remove water, the catalyst loading was 0.1% (i.e., 0.025% Cu). The solid shale was transferred into the closed reactor, after the reactor was sealed, the residual air was purged with pure nitrogen. Prior to pyrolysis, the system was pressurized to 2 MPa with nitrogen. The reactor was then heated to 450 °C at 10 °C/min and maintained for 3 h. During the pyrolysis process, the reaction pressure reached ∼7 MPa, which was attributed to the continuous accumulation of oil and gas generated during pyrolysis. The generated shale oil and hydrocarbon gases were harvested for further analysis after pyrolysis was finished. The shale oil yield was calculated as the absolute weight of the collected product oil divided by the initial weight of the raw oil shale loaded into the reactor.

### 2.5 Analysis of Pyrolysis Products

The composition of the shale gas generated from shale pyrolysis was further analyzed by Agilent 7890B gas chromatography, in which FID (Flame Ionization Detector) and TCD (Thermal Conductivity Detector) detectors were coupled with a DM-PLOT 5A molecular sieve column, a Porapak column and an HP-AL/S capillary column. Analysis by simulated distillation was performed on an Agilent 6890N chromatograph according to ASTM D 7169. The contents of C, O, and H in the product oil were determined using a Thermo Flash 2000 organic elemental analyzer. For S and N elements, an RPP-7000SN analyzer was used. The total sulfur was determined by ultraviolet fluorescence spectrometry, whereas total nitrogen was quantified by chemiluminescence detection. The product oil was analyzed by GC-MS; separation was performed on an Agilent 19091S-436 HP-5 capillary column. The mass spectrometer was operated in full-scan acquisition mode, covering a mass range of 35–500 Da at a scanning frequency of 1 scan per second.

## 3 Results and Discussion

### 3.1 Catalyst Characterization

Figure S1 shows the morphological characteristics of the Cu-TEPA catalyst. The structure of Cu-TEPA was investigated by XRD; the pattern (Figure 2a) exhibits broad peaks, indicative of its amorphous nature. The FT-IR spectra (Figure 2b) showed a broad absorption peak from 3300 to 3200 cm^–1^, ascribed to the stretching modes of −NH– and −NH_2_. A sharp peak near 2900 cm^–1^ is assigned to the symmetric and asymmetric stretching vibrations of the CH_2_ groups. A relatively strong absorption peak around 1600 cm^–1^ is attributed to the scissoring vibrations of primary amine (−NH_2_−) and the bending vibrations of secondary amine (−NH−). The absorption band located at 1465 cm^–1^ corresponds to the bending modes of −CH_2_–, another characteristic feature of the ligand skeleton. In the low wavenumber region (500–600 cm^–1^), the stretching vibration of the Cu–N bond is observed,^48^ confirming the coordination between Cu^2+^ and the nitrogen atoms of TEPA.

**Figure 2:**
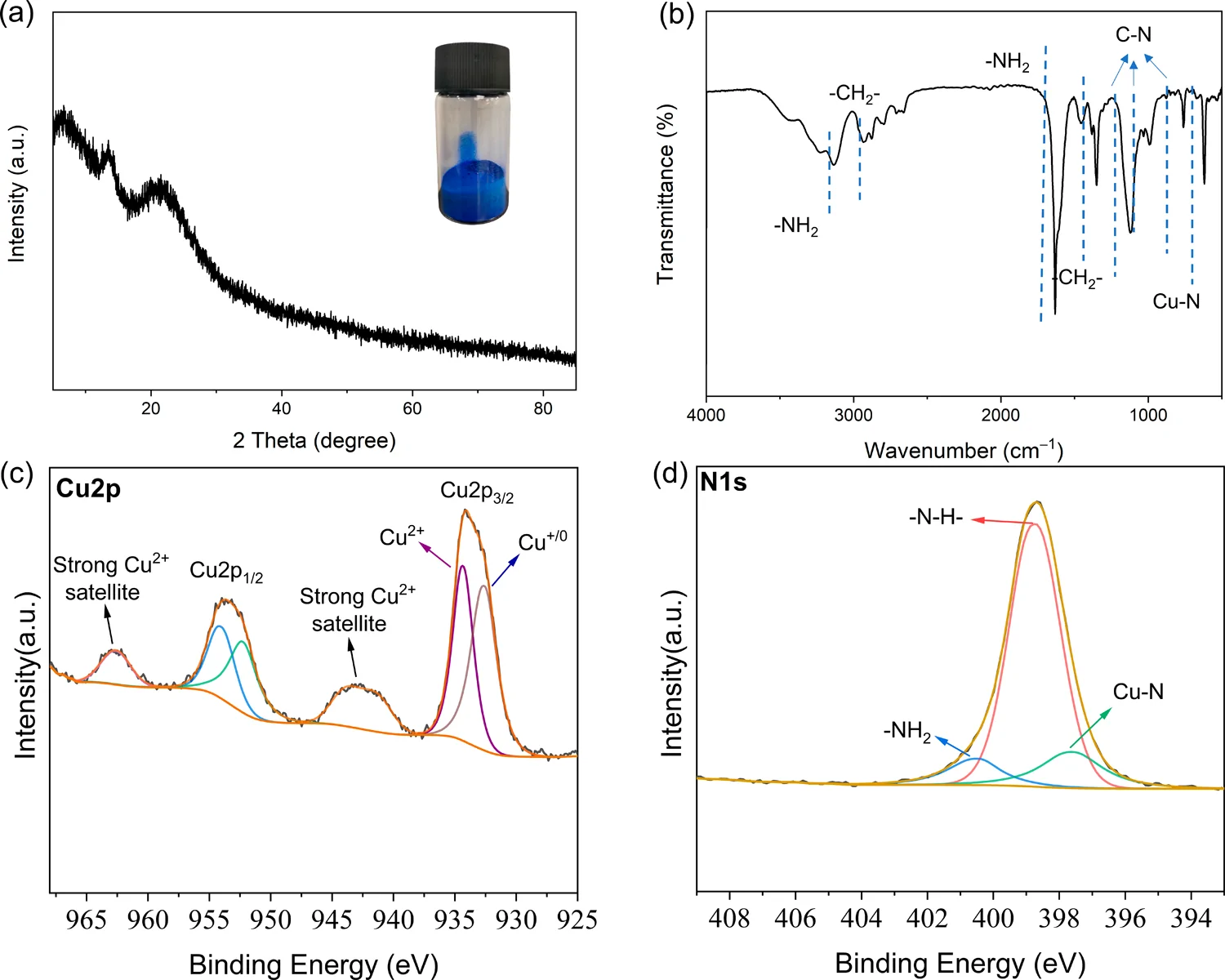
(a) XRD pattern, (b) FT-IR spectra of Cu-TEPA catalyst. XPS spectra of (c) Cu 2p, (d) N 1s on the Cu-TEPA catalyst.

Figure 2c,d presents the XPS spectra of Cu-TEPA. All peak positions were calibrated using the C 1s peak at 284.8 eV. In the Cu 2p spectrum, the main peak at 934.4 eV corresponds to Cu^2+^, which is the dominant form of copper in the material, and its characteristic peak further confirms the predominance of divalent copper. The peak at 932.6 eV is attributed to Cu^0^/Cu^+^,^49^ presumably caused by surface reduction or local changes in the coordination environment. The N 1s spectrum exhibits three distinct fitted peaks: the dominant peak centered at 398.7 eV is ascribed to −N–H– in the ligand,^50^ the peak at 400.5 eV is ascribed to terminal −NH_2_ (primary amine), and the peak at 397.7 eV is the Cu–N coordination bond,^49^ which directly verifies the successful coordination between copper and the nitrogen atoms of tetraethylenepentamine. To further elucidate the active species under actual reaction conditions, XPS analysis was conducted on the solid residue of Cu-TEPA after thermal treatment at 450 °C in the closed system (Figure S3). The Cu 2p spectrum of the residue clearly demonstrates the persistence of highly dispersed Cu^2+^ species, as evidenced by the characteristic binding energy and strong satellite peaks. Concurrently, a prominent peak corresponding to reduced Cu^+/0^ species is observed. This indicates that while the organic TEPA ligand decomposes at elevated temperatures, the remaining inorganic copper species undergo in situ phase transformations. The coexistence of Cu^2+^ and the catalytically active lower-valence Cu^+/0^ species serves as the actual catalytic centers, directly promoting kerogen pyrolysis and C–C bond cleavage at 450 °C.

Thermogravimetric analysis was performed on the Cu-TEPA catalyst. As shown in Figure S2, in the medium temperature stage (200–300 °C), the organic ligands decomposed. The remaining inorganic residues in the high temperature stage, such as CuO, may act as active centers and play a catalytic role in the oil shale pyrolysis reaction, for example, promoting the breaking of certain specific chemical bonds, affecting the distribution of pyrolysis products, lightening product oil, and increasing the proportion of gasoline fractions. Cu-TEPA catalyst exhibits suitable thermal stability for the pyrolysis reaction process in the temperature range of oil shale pyrolysis (300–600 °C), and its decomposition products may further promote pyrolysis reaction.

Furthermore, TGA analysis was conducted on raw oil shale and oil shale loaded with different contents of Cu-TEPA catalyst (as depicted in Figure 3). The pyrolysis of shale (Figure 3a) mainly consists of three stages. Below 300 °C, a minor weight loss is observed for the oil shale, which is mainly caused by the removal of moisture in the shale. Within the second stage at the range of 300–600 °C, the oil shale undergoes a significant mass loss, which mainly arises from the thermal decomposition of kerogen—a macromolecular organic matter in oil shale. In this stage, kerogen molecules crack into asphaltenes, which are further pyrolyzed into lighter product oil. This phase is crucial for the generation of shale oil and gaseous product. Above 600 °C, the third step involves the decomposition of inorganic matter, including clay minerals, carbonate minerals, sulfide minerals, and so forth.

**Figure 3:**
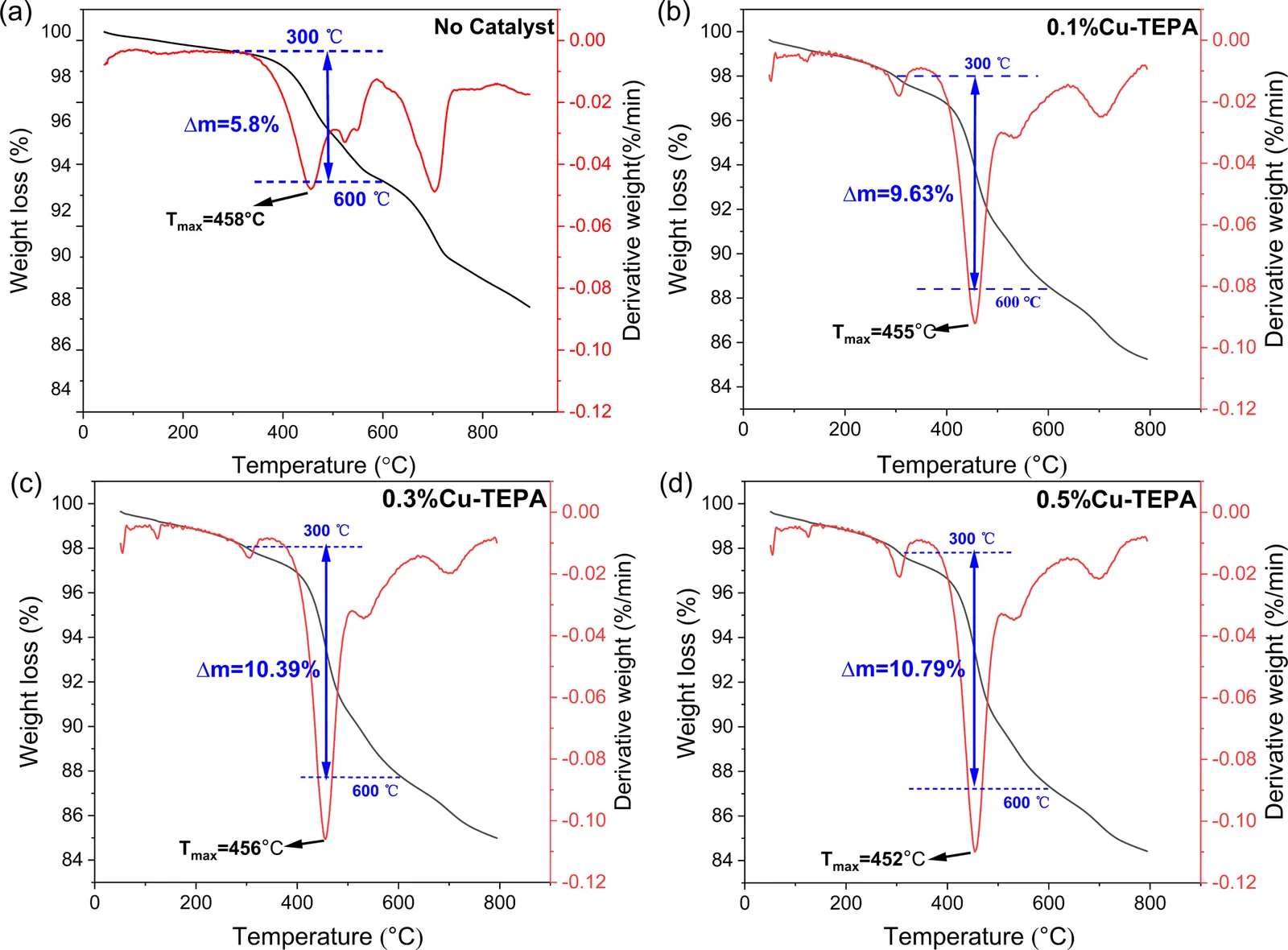
(a) TGA and Derivative thermogravimetric (DTG) curves of noncatalyzed pyrolysis of Chang 7 shale; (b–d) TGA and DTG curves of shale pyrolysis with different amounts of Cu-TEPA.

After the introduction of Cu-TEPA, the mass loss during the organic matter decomposition stage (300–600 °C) increases markedly. Notably, the sharp enhancement of catalytic performance occurs at an extremely low catalyst dosage. Compared with noncatalytic pyrolysis (Δ*m* = 5.80%), the addition of merely 0.1% Cu-TEPA substantially raises the mass loss to 9.63%, corresponding to a 66.0% increment. In contrast, further increasing the catalyst loading from 0.1 to 0.5% only leads to a slight rise in mass loss from 9.63 to 10.79%. This observation clearly demonstrates that trace amounts of Cu-TEPA (0.1%) are sufficient to trigger prominent catalytic effects, whereas additional catalyst loading delivers only marginal benefits. DTG curves reveal that the temperature corresponding to the maximum weight loss rate of kerogen decreases slightly, and the secondary decomposition stage (the cracking of asphaltenes into light hydrocarbons) shifts to lower temperatures, which corroborates the catalytic capability of Cu-TEPA. The core implication of these findings is that efficient pyrolysis can be achieved with an extremely low catalyst dosage in practical applications, which bears great significance for cost reduction and engineering implementation.

### 3.2 Analysis of Pyrolytic Behavior

#### 3.2.1 Product Yield

Figure 4 illustrates the influence of the Cu-TEPA catalyst on shale pyrolysis. Thirty g of Chang 7 shale was pyrolyzed without catalyst in the closed reactor, yielding 0.40% product oil and 6.90% pyrolysis gas. The addition of Cu-TEPA catalyst remarkably promoted the shale pyrolysis and boosted the production of shale oil and gas. Under the same reaction conditions and with only 0.1% Cu-TEPA addition (i.e., 0.025% Cu), the shale oil and gaseous product yields attained 0.53 and 7.47%, which corresponds to a 33.33% improvement in oil yield and an 8.70% elevation in total product yield relative to the noncatalytic pyrolysis (Table S4). These findings confirm the outstanding catalytic performance of Cu-TEPA in facilitating kerogen pyrolysis. However, compared to the significant yield enhancement observed in the open TGA system, the closed-system pyrolysis exhibited a lower net enhancement. This discrepancy highlights the critical impact of mass transfer and thermodynamics in pressurized environments. In the open TGA system, primary pyrolytic volatiles are continuously removed, minimizing secondary reactions. Conversely, in the closed reactor, the accumulation of oil and gas drives the internal pressure to approximately 7 MPa. Thermodynamically, this extreme pressure restrains the volume-expanding phase transformation of solid kerogen, thereby suppressing its primary decomposition.^9^ More importantly, the closed system significantly prolongs the residence time of primary liquid products (e.g., intermediate bitumen and heavy oil) in the 450 °C reaction zone. This extended thermal exposure forces the initially generated macromolecular hydrocarbons to undergo severe secondary reactions, including overcracking into noncondensable light gases and repolymerization into solid coke.^9,51^

**Figure 4:**
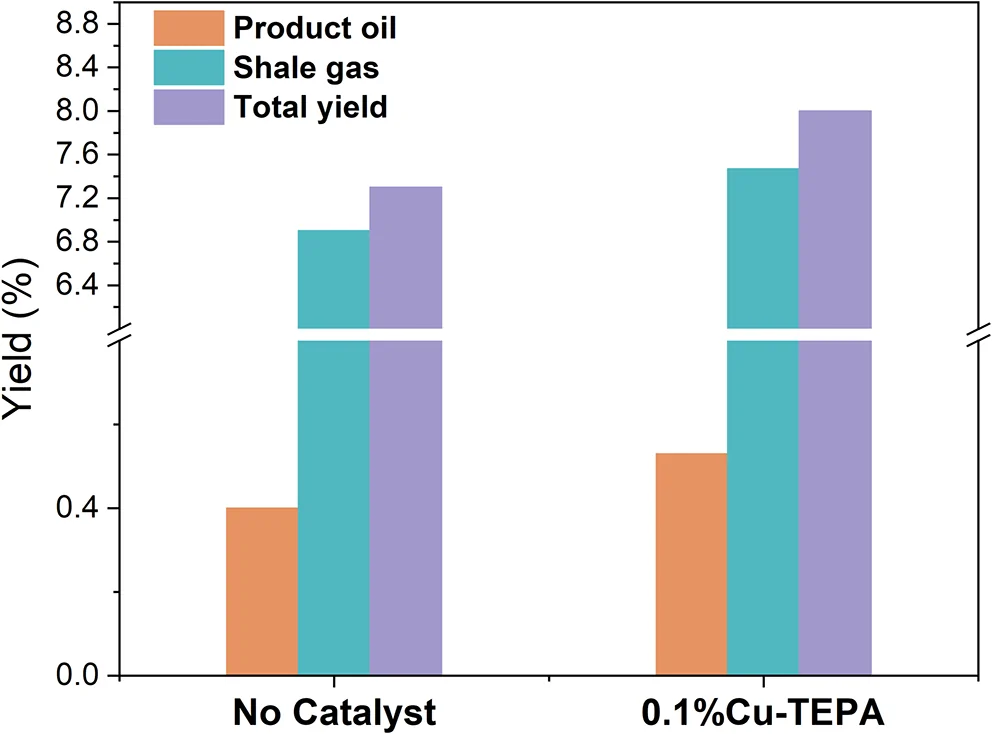
Yields of product oil and gas from noncatalytic and catalytic pyrolysis.

#### 3.2.2 Analysis of Pyrolysis Gas

(Figure 5) Composition of pyrolysis gas produced from oil shale pyrolysis with and without the Cu-TEPA catalyst. The gas products are mainly composed of H_2_, CO_2_, CH_4_, C_2_H_6_, and C_3_H_8_, together with minor amounts of C_2_H_4_, C_3_H_6_, and C_4_ hydrocarbons. Pyrolysis is dominated by free-radical reactions, so alkanes are more abundant than alkenes; furthermore, the high-pressure enclosed pyrolysis system accelerates the hydrogenation of olefins. Compared with uncatalyzed pyrolysis, slight fluctuations in H_2_ and CH_4_ contents are observed after the addition of Cu-TEPA, while the CO_2_ content remains nearly unchanged.

**Figure 5:**
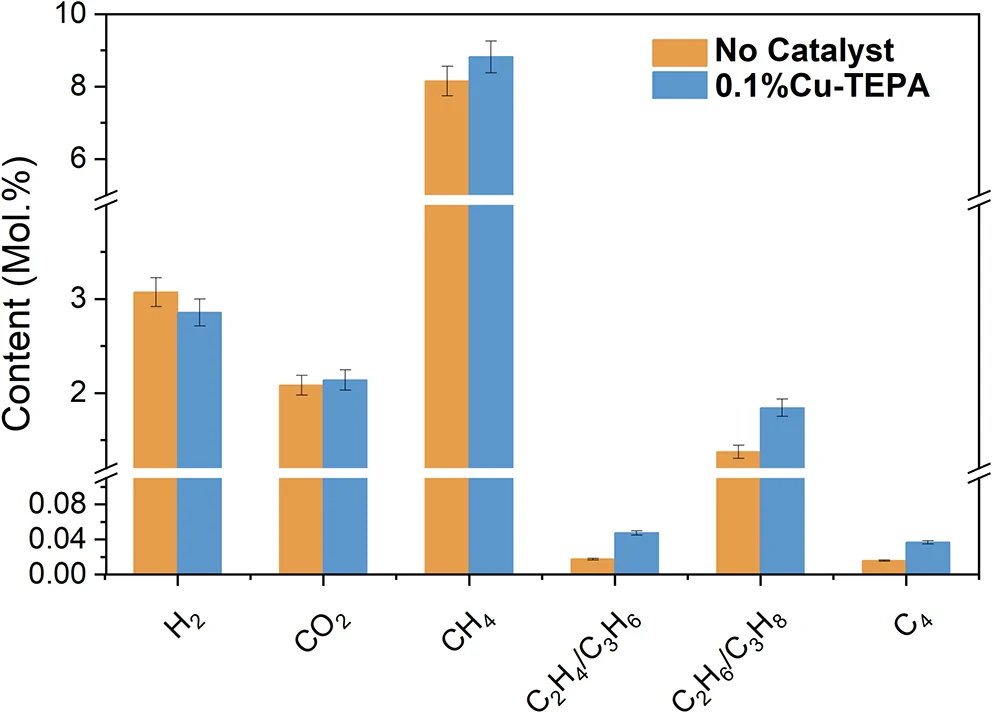
Composition of shale gas obtained from noncatalytic and catalytic pyrolysis.

#### 3.2.3 Analysis of Product Oil

To investigate whether the quality of pyrolysis-derived shale oil has been enhanced with the application of the Cu-TEPA catalyst, a comparative chemical element analysis was conducted (Figure 6a). It is well-established that the breakage of C–X (X: heteroatoms include S, N, and O) and C–C bonds in large molecular components (kerogen and asphaltenes) plays a crucial role in shale oil upgrading. After using the Cu-TEPA catalyst, the O content showed no significant change, but the S content decreased from 1.16 to 0.78% and the N content remarkably decreased from 0.89 to 0.40%. Importantly, the H/C ratio increased from 1.16 to 1.23 (Table S5), which indicates that the quality of shale oil has been significantly improved via the catalyzed pyrolysis. Furthermore, the observed product upgrading is intrinsically linked to the evolution of the copper active species during thermal processing. During pyrolysis, the decomposition of the TEPA ligand, alongside the reductive gases (e.g., H_2_ and CH_4_) generated from the initial cracking of kerogen, provides a strongly reductive environment. This in situ process facilitates the reduction of Cu^2+^ into low-valence active species, primarily Cu^+^ and Cu^0^, which is consistent with the peak located at 932.58 eV in the XPS spectrum. These in situ formed Cu^+^ sites are well-documented to possess a robust capability for driving C–C bond cleavage and hydrodenitrogenation (HDN). Consequently, the presence of these highly active low-valence copper species mechanistically accounts for the pronounced reduction in the nitrogen content and supports the enhanced light fractions and overall upgrading of the product oil.

**Figure 6:**
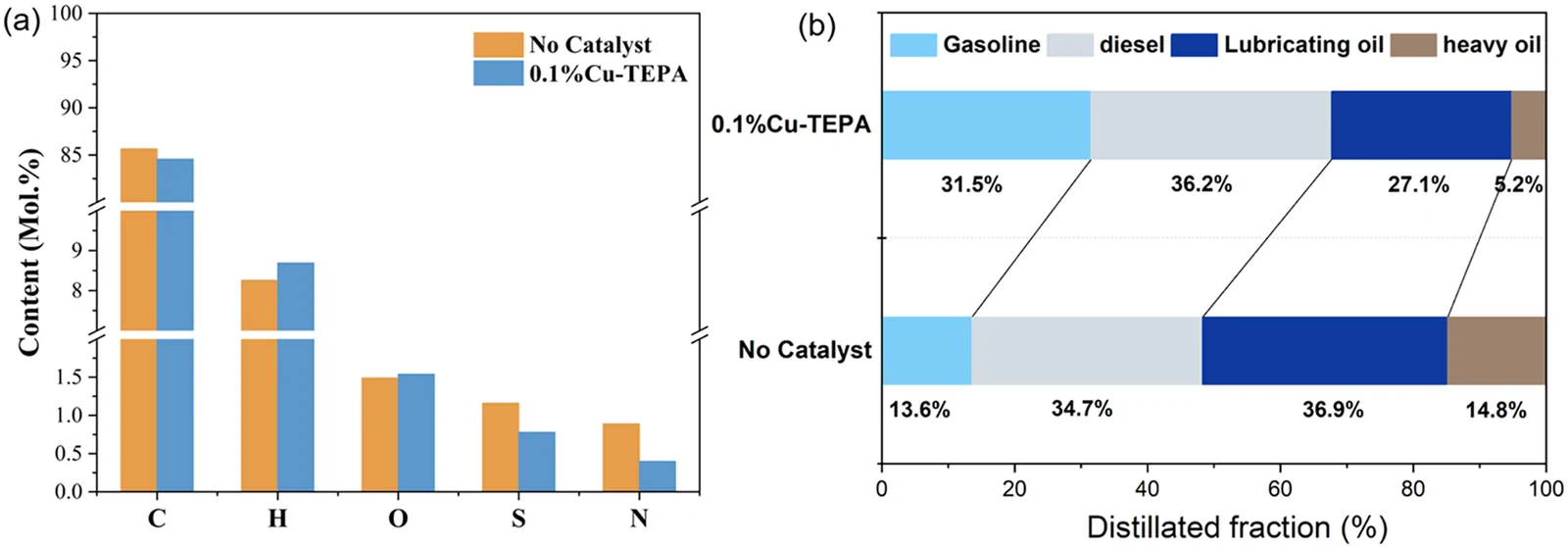
Elemental analysis (a) and simulated distillation results (b) of product oil.

The composition of product oil was characterized via the simulated distillation (Figure 6b). The oil sample is mainly composed of gasoline (IBP, initial boiling point, ∼220 °C), diesel (220∼350 °C), lubricating oil (350∼500 °C), and heavy oil (>500 °C) fractions. Cu-TEPA exerts a substantial influence on the fraction distribution of product oil, showing an obvious tendency toward lighter fractions. When Chang 7 shale was pyrolyzed without catalyst, the gasoline fraction was only 13.6%; after adding Cu-TEPA, this proportion increased significantly to 31.5%. This indicates that Cu-TEPA can strongly drive the conversion of macromolecular hydrocarbons to small-molecular gasoline during oil shale pyrolysis, greatly increasing the output of gasoline fractions and making a prominent contribution to the light-weighting of shale oil. The diesel fraction accounts for 34.7% in the case of noncatalyzed pyrolysis, and slightly increases to 36.2% after adding the catalyst. This is because diesel is in an intermediate conversion state: part of the lubricating oil is cracked to supplement diesel, while part of the diesel is further cracked into gasoline. The two effects offset each other, resulting in an insignificant change in the proportion of lighter oil. Without the catalyst, the lubricating oil fraction accounts for the highest proportion (36.9%); after the introduction of Cu-TEPA, the proportion drops to 27.1%. This indicates that the catalyst accelerates the cracking and conversion of lubricating oil fractions, transferring them to lighter fractions such as gasoline and diesel. The heavy oil fraction accounts for 14.8% in the case of noncatalyzed pyrolysis, and sharply decreases to 5.2% after adding the catalyst, with a drop of 9.6%. These results further support the elemental analysis and reveal that Cu-TEPA could significantly improve the quality of product oil.

#### 3.2.4 GC-MS Analysis

A comparison of the GC-MS total ion chromatograms of product oil samples is shown in Figure 7. Both chromatograms show intense signals in the C10–C16 region, indicating that compounds within this carbon chain range are the primary products. However, with the application of Cu-TEPA, the signals became more concentrated in the C10–C15 region, while those corresponding to C16 and longer chains were markedly weakened. This demonstrates that the catalyst facilitates the conversion of heavier carbon chains into lighter ones, thereby increasing the yield of light oil fractions. Notably, the peaks in the C10 and C11 regions became more prominent when the catalyst was used, indicating that Cu-TEPA promotes the enrichment of lower-carbon-number components, simplifies the oil composition, significantly increases the proportion of light distillates, and enhances the overall upgrading of product oil.

**Figure 7:**
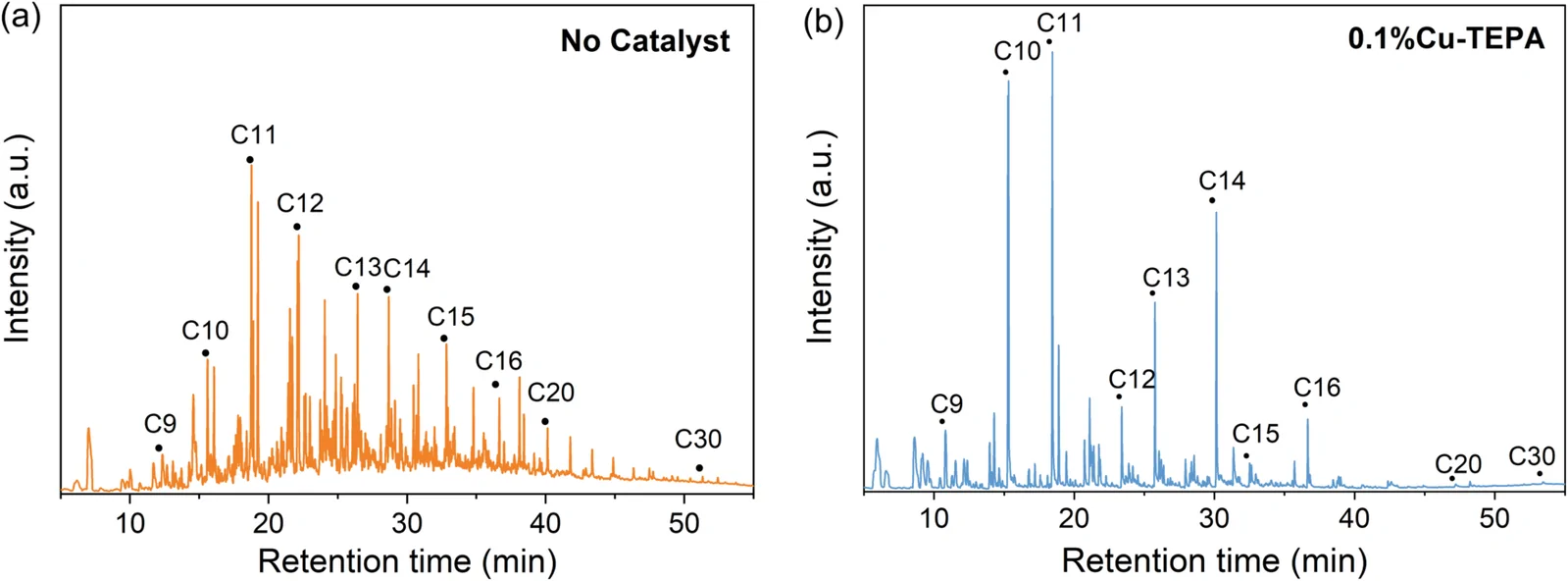
GC-MS ion spectrum of product oil obtained from pyrolysis of Chang 7 shale without (a) or with (b) the catalyst.

The impact of Cu-TEPA catalyst on the organic chemical composition of product oil during pyrolysis was investigated (as shown in Figure 8). The findings reveal that the addition of the catalyst increases the percentages of alkanes and aromatics while simultaneously reducing the content of heteroatomic compounds. Furthermore, the catalyst promoted the formation of lower carbon number components and suppressed the generation of heavier fractions. These results demonstrated the effective upgrading capability of the Cu-TEPA catalyst, which efficiently enhances the yield of light oil products and improves the overall quality.

**Figure 8:**
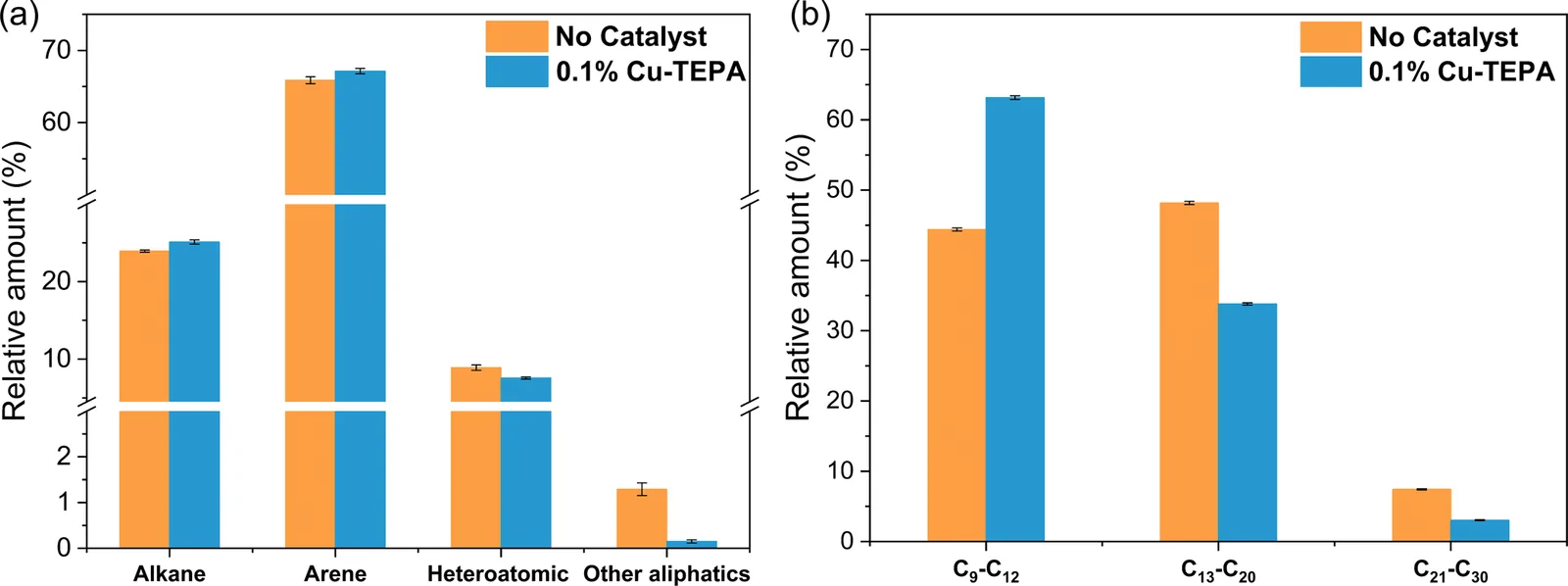
Effect of the Cu-TEPA catalyst on the distribution characteristics (a) and carbon number distribution (b) of product oil.

#### 3.2.5 Semicoke Analysis

Semicoke is the solid residue after oil shale pyrolysis, mainly composed of fixed carbon, inorganic minerals, and a small amount of incompletely decomposed organic matter. TGA and EPR characterizations of semicoke enable us to reveal the conversion extent of kerogen as well as free-radical concentration inside semicoke residues. As shown in the thermogravimetric (TG) curves of semicoke residues obtained from pyrolysis (Figure 9), semicoke collected after pyrolysis with Cu-TEPA exhibited reduced mass loss in the organic-matter decomposition stage (300–600 °C), compared with semicoke from catalyst-free pyrolysis. This indicates that Cu-TEPA has already exerted its effect during the oil shale pyrolysis stage, promoting the decomposition of kerogen in oil shale and converting more organic matter into oil and gas products. Consequently, the content of residual pyrolyzable organic matter/fixed carbon in the finally generated semicoke is significantly reduced. It can be seen from the derivative thermogravimetric (DTG) curve that the main thermal conversion interval of both groups of semicoke is concentrated around 500 °C. However, after the addition of Cu-TEPA, the peak value of the thermal conversion rate of semicoke becomes gentler, which further confirms the reduction of convertible substances in the semicoke.

**Figure 9:**
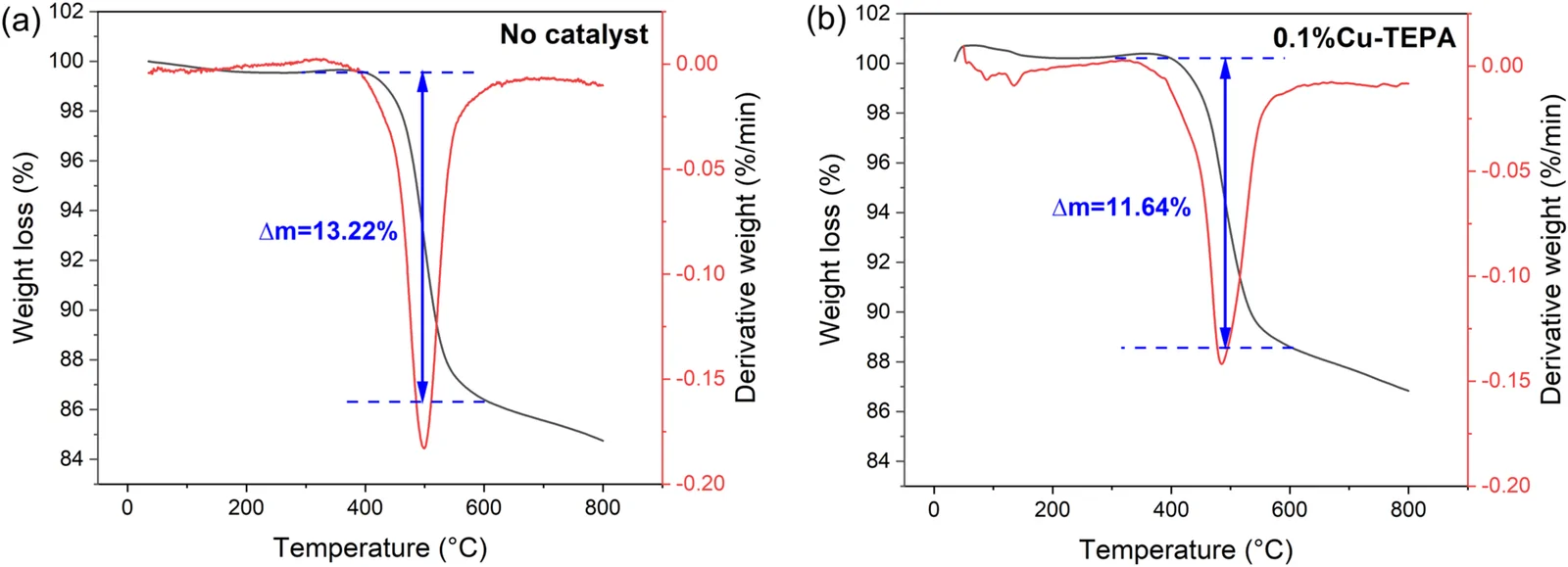
TGA and DTG curves of semicokes obtained from pyrolysis of Chang 7 shale without (a) or with (b) the catalyst.

Figure 10 illustrates the EPR spectra of the semicokes generated under noncatalytic and catalytic pyrolysis conditions. Both samples display analogous EPR spectral profiles; however, the addition of Cu-TEPA significantly influences the intensity of the EPR peak, indicating the reduction of free-radical concentration. This phenomenon probably arises from two mechanisms: (1) the catalyst enhances radical consumption in reactions that generate product oil and gas, thereby attenuating the paramagnetic signal; and (2) it accelerates kerogen cracking, which reduces the overall radical population as the primary organic carrier decomposes. The *g*-value shift (Table S6) from 2.00374 to 2.00598 reflects strong spin–orbit coupling, primarily attributed to the paramagnetic Cu^2+^ ions retained in the residue.^52^

**Figure 10:**
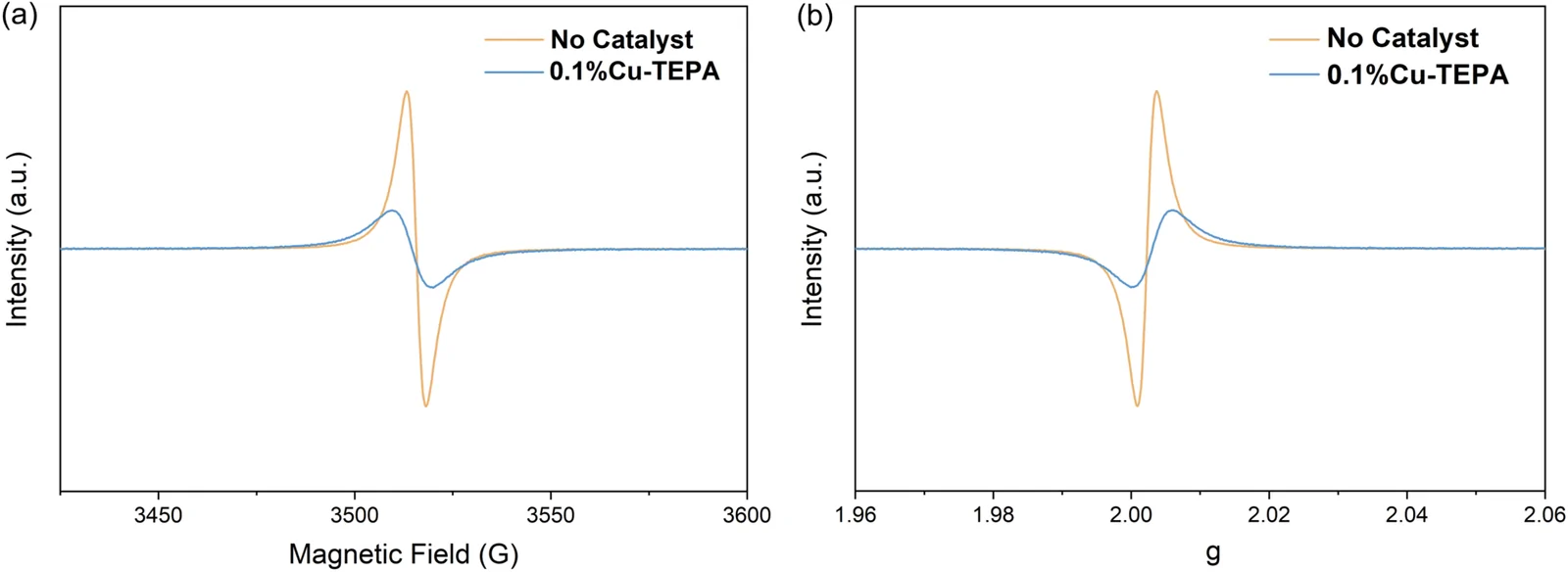
EPR spectra obtained from semicoke samples with and without 0.1% Cu-TEPA catalyst. (a) Spectra plotted versus magnetic field (G); (b) spectra presented against g-factor.

## 4 Conclusions

In summary, the copper-based catalyst Cu-TEPA exhibits a remarkable catalytic performance for oil shale pyrolysis. The results reveal that the catalyst improves the oil recovery, and the yield of product oil increases from 0.40% under noncatalytic pyrolysis to 0.53% under catalytic pyrolysis, representing a 33.33% enhancement. In addition, upon loading Cu-TEPA, the content of light-oil fractions (gasoline and diesel) in product oil was significantly increased from 48.3 to 67.7%. This phenomenon is further supported by the carbon number distribution and H/C ratio in the oil products. Furthermore, the catalyst also displays remarkable efficiency in heteroatom removal; the markedly decreased sulfur and nitrogen levels signify efficient upgrading of the shale oil. These salient findings highlight the importance of catalysts in oil shale pyrolysis and demonstrate considerable potential for practical application of Cu-TEPA in the in situ conversion of oil shale.

## Supplementary Material



## Data Availability

Data will be made available on request.

## Notes

SEM image of Cu-TEPA (Figure S1); TGA curve and DTG profile of Cu-TEPA catalyst (Figure S2); XPS spectra of the Cu-TEPA residue collected after thermal treatment under pyrolysis conditions (Figure S3); The coal quality analysis of Chang 7 shale (Table S1); The property parameters of Chang 7 shale (Table S2); The elemental content of Chang 7 shale (Table S3); Yields of product oil and gas (Table S4); Elemental analysis of product oil obtained from noncatalytic and catalytic pyrolysis (Table S5). EPR results of semicoke samples (Table S6) (PDF)
