# Supplementary material for: In Situ Pyrolysis of Oil Shale with the Copper-Amine Complex as an Efficient Catalyst
Source: ACS Omega. 2026 Sep 25;11(39):59522–31. doi: 10.1021/acsomega.6c05797 (PMC13647371; doi:10.1021/acsomega.6c05797)
Supplement: Supplementary file 1 [file ao6c05797_si_001.pdf]

Supporting Information

In-situ Pyrolysis of Oil Shale with Copper-amine Complex as Efficient Catalyst

Lianhua Hou[a]†, Chen Wang[b]†, Jing Chen[b], Bo Sun[b], Ziyi Xu[a], Baojian Shen[b], Chunming Xu[b], Xiaochun Zhu[b]**

[a]Research Institute of Petroleum Exploration & Development, PetroChina, 100083 Beijing, P. R. China

[b]State Key Laboratory of Heavy Oil Processing, College of Chemical Engineering and Environment, China University of Petroleum Beijing, 102249 Beijing, P. R. China

*Corresponding author. Email: houlh@petrochina.com.cn; xzhu@cup.edu.cn

†These authors contributed equally to this work

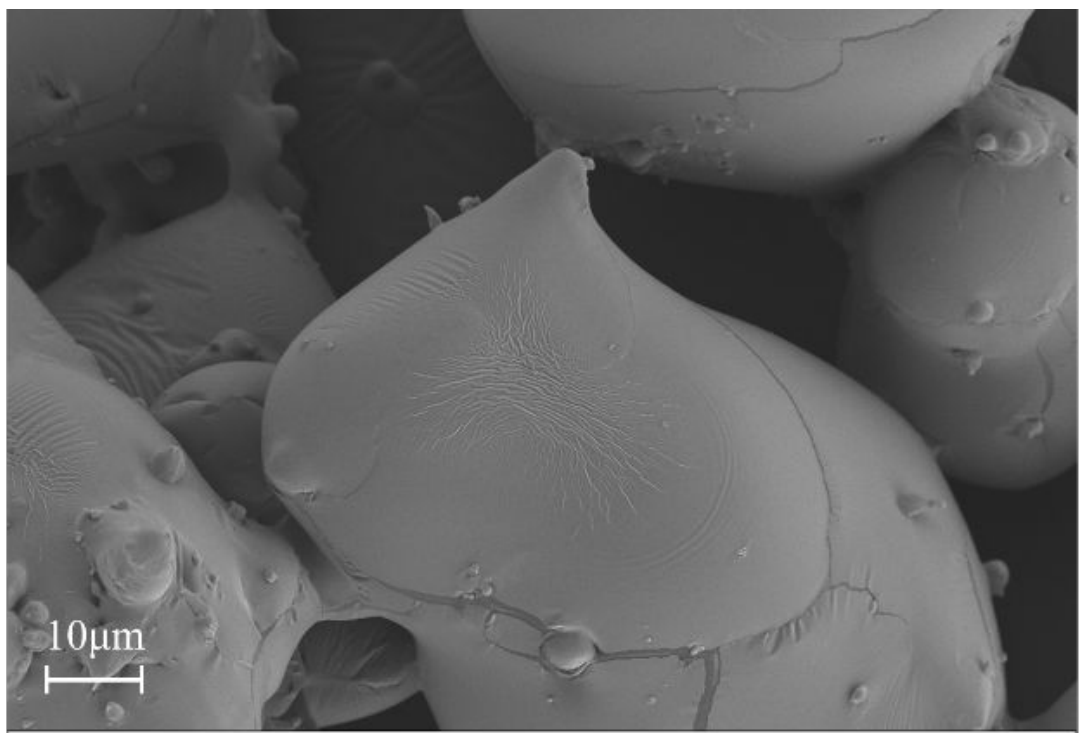

Figure S1. SEM image of Cu-TEPA.

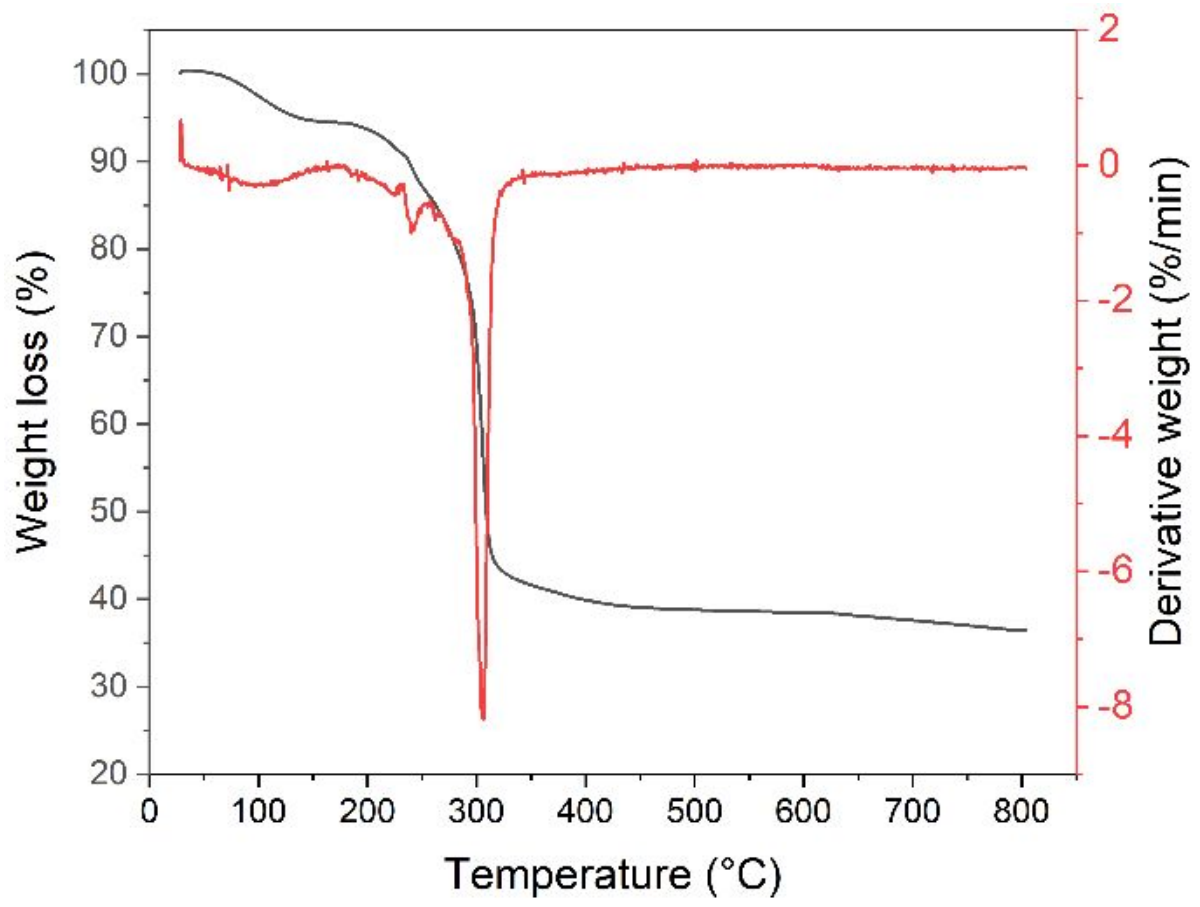

Figure S2. TGA curve and DTG profile of Cu-TEPA catalyst.

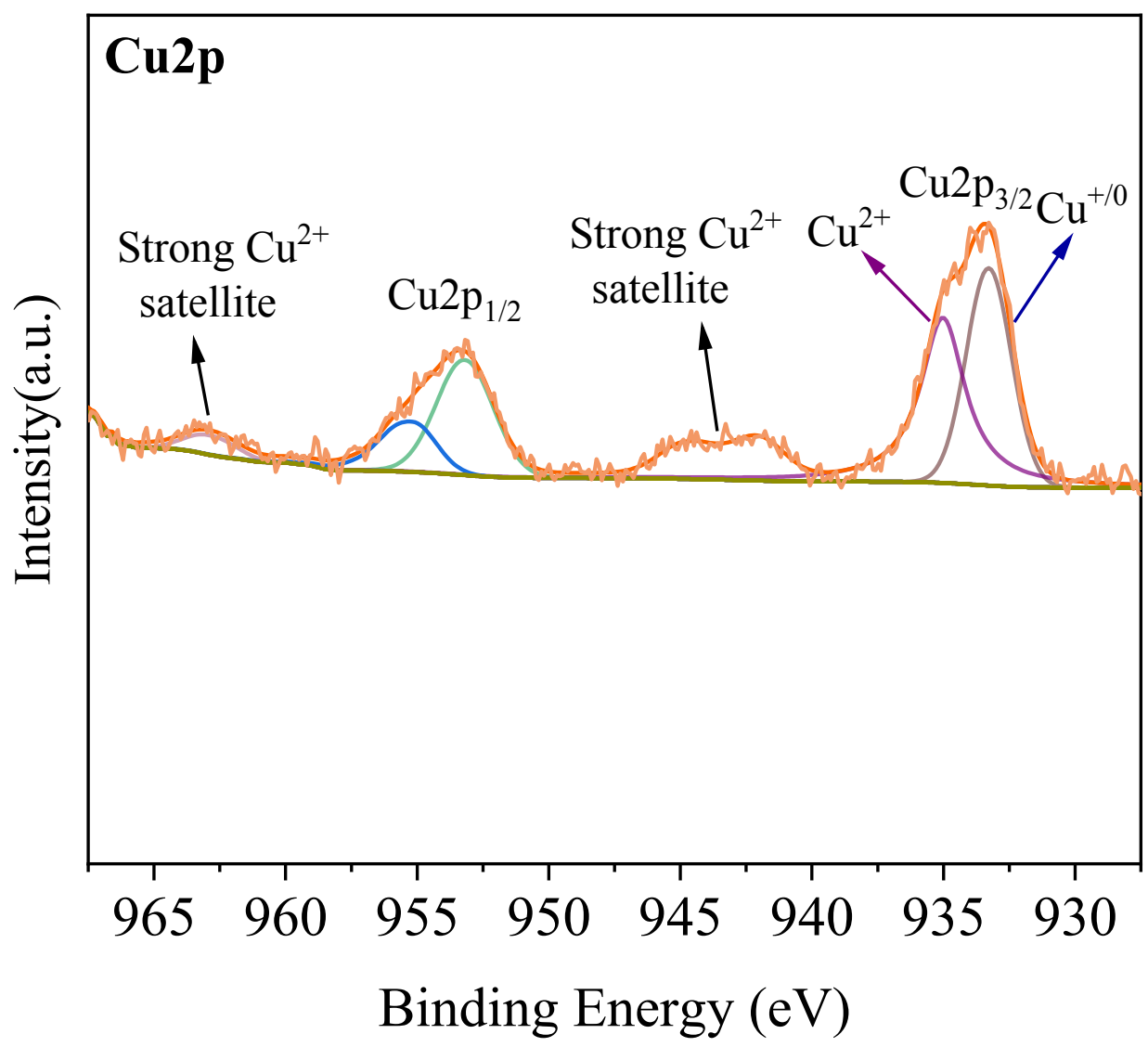

Figure S3. XPS spectra of the Cu-TEPA residue collected after thermal treatment under pyrolysis conditions.

Table S1 The coal quality analysis of Chang 7 shale.

M_{ad} [a] (%)	A_{ad} [b] (%)	V_{ad} [c] (%)	CRC [d] (1~8)	FC_{ad} [e] (%)
0.55	83.57	12.02	2	3.86

[a] M_{ad}: Moisture on air-dried basis;

[b] A_{ad}: Ash on air-dried basis;

[c] V_{ad}: Volatile matter on air-dried basis;

[d] CRC: Coke Residue Characteristics;

[e] FC_{ad}: Fixed carbon on air-dried basis.

Table S2 The property parameters of Chang 7 shale.

TOC [a] (%)	T_{max} (℃)	S1+S2 [b] (mg HC/g rock)	PC (%)	HI [c]
17.10	439.17	69.17	5.78	467.85

[a] TOC: Total Organic Carbon;

[b] S1+S2: It refers to the maximum mass potential of organic matters such as kerogen in oil shale to be converted into hydrocarbons under pyrolysis (or catalytic pyrolysis) conditions. It is positively correlated with the organic carbon (OC) content in oil shale, and is usually determined by the Rock-Eval Pyrolysis method, with the corresponding key parameter being the sum of S_1 (free hydrocarbons) and S_2 (pyrolytic hydrocarbons).

[c] HI: Hydrogen Index.

Table S3 The elemental content of Chang 7 shale.

Element content (wt%)					
C	H	O	N	S	Si
9.507	1.446	1.814	1.047	8.088	78.098

Table S4 Yields of product oil and gas.

Sample	No Catalyst	0.1%Cu-TEPA
Product oil, %	0.40	0.53
Shale gas, %	7.30	7.84
Total yield, %	7.70	8.37
Improvement effect of extraction rate, %	–	8.70

Table S5 Elemental analysis of product oil obtained from non-catalytic and catalytic pyrolysis.

	C	H	O	S	N	H/C ratio
No Catalyst	85.67	8.26	1.49	1.16	0.89	1.16
0.1%Cu-TEPA	84.58	8.69	1.54	0.78	0.40	1.23

Table S6 EPR results of semi-coke samples.

Sample	No catalyst	0.1%Cu-TEPA
Free radical, spins/g	2.058×10^{18}	1.675×10^{18}
g	2.00374	2.00598
